# Body mass index as an independent risk factor for inferior vena cava resection during thrombectomy for venous tumor thrombus of renal cell carcinoma

**DOI:** 10.1186/s12957-019-1560-5

**Published:** 2019-01-15

**Authors:** Bin-Shuai Wang, Run-Zhuo Ma, Yu-Qing Liu, Zhuo Liu, Li-Yuan Tao, Min Lu, Guo-Liang Wang, Shu-Dong Zhang, Jian Lu, Lu-Lin Ma

**Affiliations:** 1 Department of Urology, Peking University Third Hospital, Beijing, China; 2 Research Center of Clinical Epidemiology, Peking University Third Hospital, Beijing, China; 3 Department of Pathology, Peking University Third Hospital, Beijing, China

**Keywords:** Renal cell carcinoma, Venous tumor thrombus, Thrombectomy, Vascular resection, Risk factor

## Abstract

**Objective:**

To define preoperative clinical and radiographic risk factors for the need of inferior vena cava (IVC) resection in patients with renal cell carcinoma (RCC) and IVC tumor thrombus.

**Methods:**

We reviewed data of 121 patients with renal cell carcinoma and venous tumor thrombus receiving radical nephrectomy and thrombectomy at our institution between 2015 and 2017, and 86 patients with Mayo I–IV level tumor thrombus were included in the final analysis. Clinical features, operation details, and pathology data were collected. Preoperative images were reviewed separately by two radiologists. Univariable and multivariable logistic regression analyses were applied to evaluate clinical and radiographic risk factors of IVC resection.

**Results:**

Of the 86 patients, 44 (51.2%) received IVC resection during thrombectomy. In univariate analysis, we found that body mass index (BMI) (odds ratio [OR] = 1.22, *P* = 0.003), primary tumor diameter (OR = 0.84, *P* = 0.022), tumor thrombus width (OR = 1.08, *P* = 0.037), tumor thrombus level (OR = 1.57, *P* = 0.030), and IVC occlusion (OR = 2.67, *P* = 0.038) were associated with the need for resection of the IVC. After adjusting for the other factors, BMI (OR = 1.18, *P* = 0.019) was the only significant risk factor for IVC resection. Multivariable analysis in Mayo II–IV subgroups confirmed BMI as an independent risk factor (OR = 1.26, *P* = 0.024). A correlation between BMI and the width (Pearson’s correlation coefficient [PCC] = 0.27, *P* = 0.014) and length (PCC = 0.23, *P* = 0.037) of the tumor thrombus was noticed.

**Conclusion:**

We identified BMI as an independent risk factor for IVC resection during thrombectomy of RCC with tumor thrombus in a Chinese population. More careful preoperative preparation for the IVC resection and/or reconstruction is warranted in patients with higher BMI.

## Introduction

Renal cell carcinoma (RCC) represents 2–3% of all cancers [1]. In the USA, RCC represents 5% of annual new cancer cases and is the third most common cancer in the urinary system [2]. In China, the incidence rate of RCC is approximately 2% of adult malignant tumors, ranking second in urological malignancies, and the incidence rate keeps rising each year [3]. One of the unique features of RCC is venous tumor thrombus (VTT) formation, with an incidence varying from 4 to 10% among all cases [4]. The VTT could migrate from the renal vein to the inferior vena cava (IVC) and even to the right atrium.

Studies have reported that RCC with VTT leads to a 1-year disease-specific survival of 29% among untreated patients. After thrombectomy, the 5-year survival rate can increase to 40–65% [5]. Radical nephrectomy combined with thrombectomy is the only current potential curative method [6]. The survival rate of patients with tumor thrombus was better in those receiving both nephrectomy and tumor thrombectomy compared to patients receiving nephrectomy alone [7].

During thrombectomy, if the tumor was found with any invasion into the IVC wall, partial or circumferential resection of the IVC is preferred [8]. In this case, the risk of the surgery is generally high, as IVC resection is generally determined during the operation, without careful preoperative preparation. Preoperative prediction for the evaluation of IVC resection is required for treatment planning and patient counseling.

Previous studies have reported several radiographic predictors of the evaluation for IVC resection [9–12]. However, some of these studies are limited by the relatively small size of their cohort [9, 10], while others have focused on radiographic features instead of clinical characteristics [11, 12].

Therefore, the objective of our study was to define preoperative clinical and radiographic risk factors for IVC resection prediction.

## Methods and materials

### Patients

**Fig. 1:**
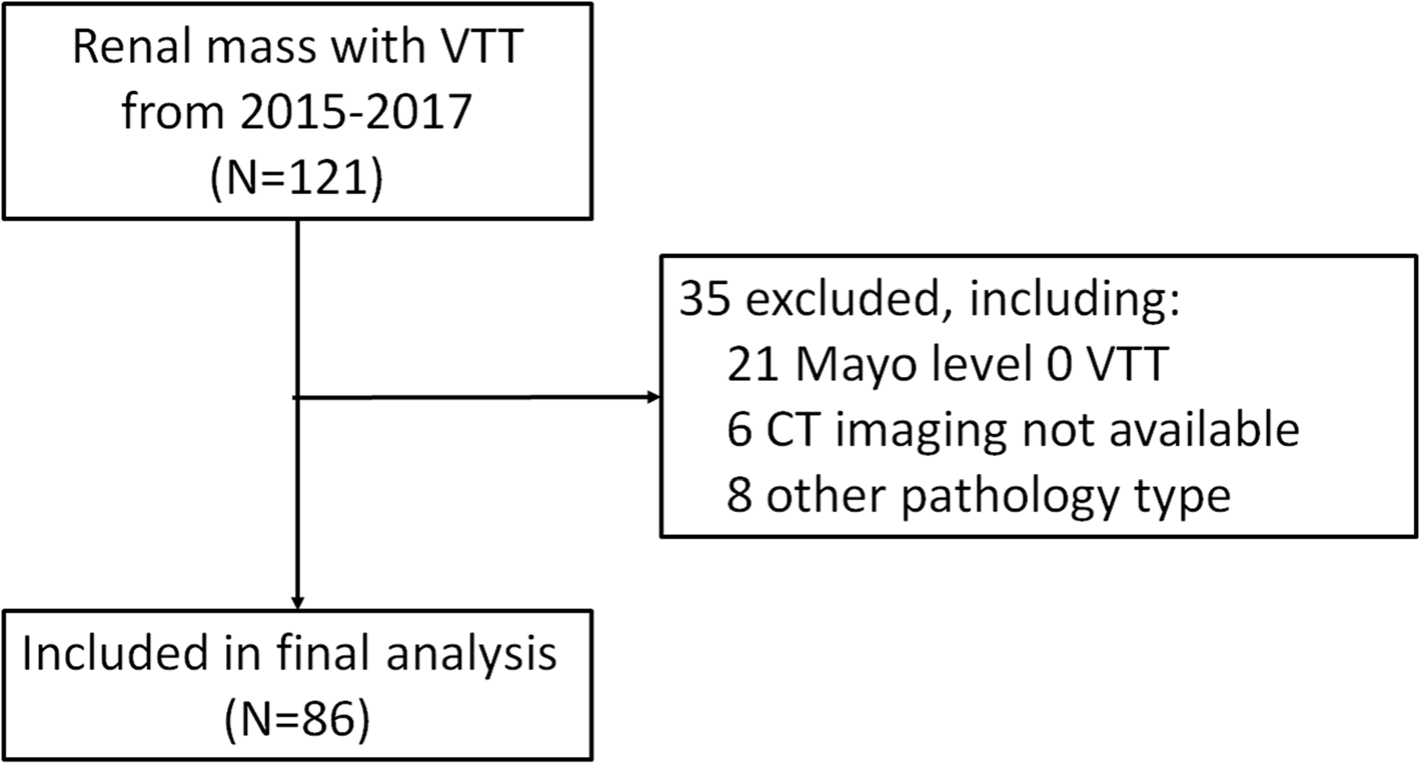
Summary of our study cohort and flow chart of exclusion criteria

After obtaining institutional review board approval, we reviewed a total of 121 cases with renal mass and VTT from January 2015 to December 2017 who received nephrectomy and thrombectomy in our institution. Exclusion criteria included (a) level 0 venous tumor thrombus (Mayo classification) [13], (b) incomplete clinical or radiographic image data, and (c) pathology type other than renal cell carcinoma. Ultimately, a total of 86 cases were included for analysis (Fig. 1).

### Clinical and radiographic features

We collected clinical features, including age, gender, comorbidities, laterality, body mass index (BMI), serum creatinine (SCr), ASA score, nodal and metastasis status, and pathologic features.

**Fig. 2:**
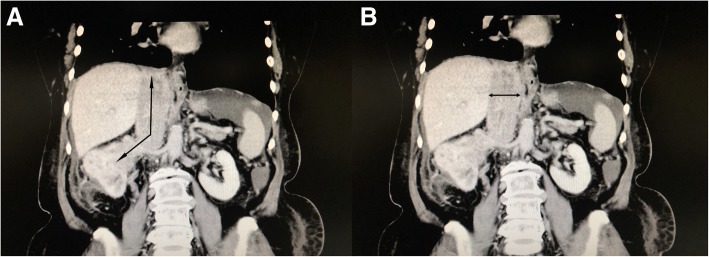
Measurement of the length and width of IVC tumor thrombus. **a** The length is the sum of the tumor thrombus in the renal vein and in the IVC. **b** The maximum width of the tumor thrombus is measured in the coronary plane

Preoperative MRI or CT data were reviewed by two radiologists blind to patients’ surgery information. We recorded whether the tumor thrombus totally occluded IVC by whether contrast medium could pass the IVC during arterial/venous phase. The length and width of the tumor thrombus were also measured (Fig. 2). In brief, the length of the tumor thrombus was measured by the sum of the length in the renal vein and the IVC. The maximum diameter of the tumor thrombus was measured in the coronary plane. The diameter of the IVC was measured at the superior level of the diaphragm. Then, the cohort was grouped by the ratio of the thrombus width to the IVC width. The cases with the ratio of < 2/3, 2/3–1, and > 1 were classified into three groups.

### Surgical approaches and outcomes of interest

The surgical approach of IVC tumor thrombectomy in our institution was described previously [14, 15]. Briefly, resection of IVC was generally determined intraoperatively. If the tumor thrombus did not grossly invade the IVC wall and could be resected integrally, thrombectomy followed by cavorrhaphy with running suture was performed. If the tumor thrombus grossly invaded the IVC wall, vein resection was undertaken to ensure at least 1-mm negative margin. After vein resection, if the remaining IVC lumen was compromised by more than half of the original diameter [16, 17], the reconstruction was performed using an autogenous graft patch (e.g., ipsilateral gonadal vein). In some cases, where the VTT was accompanied by the distal bland thrombus or the VTT circumferentially invaded the IVC, complete circumferential resection of the IVC was needed. In this situation, the left kidney could be left alone without renal vein reconstruction because of abundant collateralization, while the right kidney required renal vein reconstruction in order to achieve sufficient blood reflux. The primary outcome of interest was whether the IVC was resected during surgery. We defined any partial or segmental resection of the IVC during surgery as the endpoint of observation indicators.

### Statistical analysis

Categorical variables were summarized with percentages, and continuous variables were summarized with medians and interquartile ranges (IQRs). Chi-squared tests and two-sample *t* tests were applied for comparisons between groups. Univariate logistic regression analysis was used to analyze risk factors for the need of IVC resection, then significant factors were included in subsequent multivariate analysis. The results were summarized with odds ratios and 95% confidence intervals (CIs). The correlation between two continuous variables was calculated by Pearson’s correlation coefficient. The statistical tests were performed with SPSS 24.0 (IBM Inc., Chicago, IL, USA). All tests were two-sided, and *P* values < 0.05 were considered to be statistical significance.

## Results

Clinical and radiographic features of our cohort are shown in Table 1. Among the 86 patients, 44 (51.2%) received IVC resection. These patients were more likely to have higher BMI (23.9 kg/m^2^ vs. 22.0 kg/m^2^, *P* = 0.001), smaller diameter of renal mass (8.3 cm vs. 9.3 cm, *P* = 0.016), wider tumor thrombus in IVC (21.5 cm vs. 21.0 cm, *P* = 0.032), higher VTT levels (*P* = 0.048), and a higher percentage of IVC occlusion (45.5% vs. 23.8%, *P* = 0.035).

**Table 1:** Comparison of clinical and pathologic features by need of resection of IVC

Feature	In total *N* = 86	No. of IVC resection *N* = 42	IVC resection *N* = 44	*P* value
Median (IQRs)				
Age, years	61.0 (53.8–67.3)	60.5 (53.8–68.0)	61.0 (53.3–67.0)	0.383
BMI, kg/m^2^	23.1 (21.1–26.1)	22.0 (19.4–24.3)	23.9 (22.3–27.7)	0.001
Preoperative SCr, μmol/L	97.5 (83.5–113.0)	92.0 (78.3–113.8)	102.0 (89.0–112.8)	0.074
Tumor diameter, cm	8.4 (6.9–10.4)	9.3 (7.1–11.3)	8.3 (5.8–10.0)	0.016
TT width, cm	22.0(12.8–27.3)	21.0 (18.0–25.0)	24.5 (19.3–28.8)	0.032
*N* (%)				
Sex				
Male	61 (70.9)	26 (61.9)	35 (79.5)	0.072
Female	25 (29.1)	16 (38.1)	9 (20.5)	
Side				
Left	21 (24.4)	9 (21.4)	12 (27.3)	0.528
Right	65 (75.6)	33 (78.6)	32 (72.7)	
ASA score				
1	4 (4.7)	3 (7.1)	1 (2.3)	0.113
2	67 (77.9)	35 (83.3)	32 (72.7)	
3	15 (17.4)	4 (9.5)	11 (25.0)	
cN stage				
cN0	41 (47.7)	22 (52.4)	19 (43.2)	0.393
cN1	45 (52.3)	20 (47.6)	25 (56.8)	
cM stage				
cM0	58 (67.4)	25 (59.5)	33 (75.0)	0.126
cM1	28 (32.6)	17 (40.5)	11 (25.0)	
Mayo classification				
1	26 (30.2)	18 (42.9)	8 (18.2)	0.048
2	26 (30.2)	12 (28.6)	14 (31.8)	
3	21 (24.4)	6 (14.3)	15 (34.1)	
4	13 (15.1)	6 (14.3)	7 (15.9)	
Width of thrombus/width of IVC				
≤ 2/3	33 (38.4)	20 (47.6)	13 (29.5)	0.225
2/3–1	38 (44.2)	16 (38.1)	22 (50.0)	
>1	15 (17.4)	6 (14.3)	9 (20.5)	
IVC occlusion				
Yes	30 (34.9)	10 (23.8)	20 (45.5)	0.035
No	56 (65.1)	32 (76.2)	24 (54.5)	
Pathology type				
Clear cell	68 (79.1)	36 (85.7)	32 (72.7)	0.139*
Papillary	12 (14.0)	3 (7.1)	9 (20.5)	
Chromophobe	1 (1.2)	0	1 (2.3)	
Ewing’s sarcoma	1 (1.2)	1 (2.4)	0	
Squamous carcinoma	1 (1.2)	0	1 (2.3)	
Unclassified	3 (3.5)	2 (4.8)	1 (2.3)	
Furman’s classification				
1–2	29 (33.7)	15 (35.7)	14 (31.8)	0.702
3–4	57 (66.3)	27 (64.3)	30 (68.2)	

*IQRs* interquartile ranges, *IVC* inferior vena cava, *TT* tumor thrombus

*Clear cell type vs. others

**Fig. 3:**
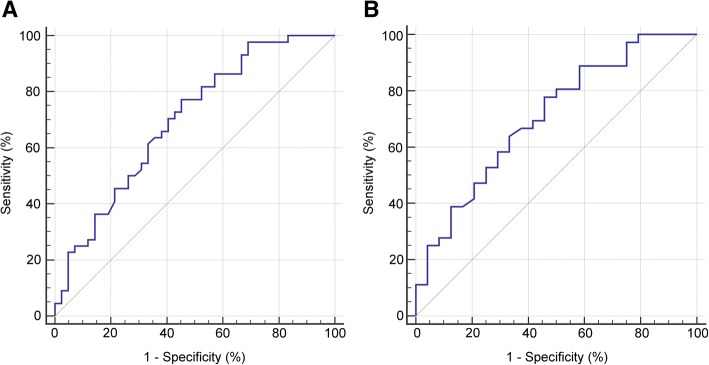
ROC curve depicting the relationship between BMI and IVC resection. **a** ROC curve of the relationship between BMI and the IVC resection in Mayo level I–IV patients. AUC = 0.70. The best cutoff value was 22.2 kg/m^2^, which achieved a sensitivity of 77.3% and a specificity of 54.8%. **b** ROC curve of the relationship between BMI and the IVC resection in Mayo level II–IV patients. AUC = 0.71. The best cutoff value was 22.4 kg/m^2^, which achieved a sensitivity of 77.8% and a specificity of 54.2%

Univariate and multivariate associations of preoperative clinical and radiographic features predicting the need for IVC resection are shown in Table 2. Univariate analysis confirmed that the aforementioned factors were significantly associated with the resection of IVC. However, in multivariate analysis, BMI was the only factor associated with IVC resection (OR = 1.18, *P* = 0.019). The receiver operating characteristic (ROC) curve depicting the relationship between BMI and resection of IVC had an area under the curve (AUC) of 0.70 (Fig. 3a). The best cutoff value was 22.2 kg/m^2^, which achieved a sensitivity of 77.3% and a specificity of 54.8%.

**Table 2:** Univariate and multivariate associations of preoperative clinical and radiographic features predicting the need for IVC resection

Feature	Univariable analysis		Multivariable analysis	
	Odds ratio (95% CI)	*P* value	Odds ratio (95% CI)	*P* value
BMI, kg/m^2^	1.22 (1.01–1.39)	0.003	1.18 (1.03–1.35)	0.019
Mayo classification	1.57 (1.03–2.41)	0.030	1.14 (0.68–1.90)	0.623
Tumor diameter, cm	0.84 (0.72–0.97)	0.022	0.86 (0.72–1.01)	0.068
TT width, cm	1.08 (1.01–1.17)	0.037	1.05 (0.96–1.14)	0.285
IVC occlusion				
No	Reference	0.038	Reference	0.403
Yes	2.67 (1.06–6.73)		1.58 (0.54–4.62)	

*TT* tumor thrombus

We noticed a significant increased possibility of IVC resection in the Mayo level II–IV cohort compared to the Mayo level I cohort (60.0% vs. 30.8%, *P* = 0.013, not shown in the tables). Therefore, we performed similar multivariate analysis in the Mayo level II–IV subgroup, and the results are shown in Table 3. BMI was still the only significant risk factor for IVC resection (OR = 1.26, *P* = 0.024). The ROC curve depicting the relationship between BMI and resection of IVC in the subgroup achieved an AUC of 0.71 (Fig. 3b). The best cutoff value was 22.4 kg/m^2^, which achieved a sensitivity of 77.8% and a specificity of 54.2%.

**Table 3:** Multivariate analysis for predictors of the need for IVC resection in the Mayo II–IV subgroup

Feature	Multivariable analysis	
	Odds ratio (95% CI)	*P* value
BMI, kg/m^2^	1.26 (1.03–1.54)	0.024
Tumor diameter, cm	0.84 (0.68–1.04)	0.107
TT width, cm	1.08 (0.97–1.20)	0.144
IVC occlusion		
Yes vs. No	1.85 (0.51–6.64)	0.347

Furthermore, we found that BMI was significantly correlated with VTT width (Pearson’s correlation coefficient [PCC] = 0.27, *P* = 0.014) and length (PCC = 0.23, *P* = 0.037). The patients comorbid with hypertension (24.8 kg/m^2^ vs. 22.5 kg/m^2^, *P* = 0.006) or diabetes (27.1 kg/m^2^ vs. 23.2 kg/m^2^, *P* = 0.012) tended to have a higher BMI.

## Discussion

The most challenging part of thrombectomy involves the resection and reconstruction of the IVC. Preoperative prediction could assist urologists in better patient counseling, reconstruction planning, and vascular surgical arranging. Therefore, we analyzed relevant clinical and radiographic features to determine the risk factors of IVC resection. In our study, 51% (44/86) of patients underwent IVC resection. We found that BMI, primary tumor diameter, tumor thrombus width, VTT level, and IVC occlusion were significantly associated with IVC resection in univariate analysis. BMI was the only independent risk factor after adjustment for the other factors.

High BMI is a well-recognized risk factor for RCC [18]. With each additional unit of BMI, the relative risk of developing RCC increases by 1.07 times [19]. Therefore, it is not surprising to find a 1.7-fold higher relative mortality risk due to RCC in the high BMI (≥ 35.0 kg/m^2^) group compared to the normal BMI (18.5–24.9 kg/m^2^) group in a population-based cohort [20]. However, for patients who have already suffered from RCC, higher BMI predicts a better overall survival and cancer-specific survival conditions [21, 22]. This phenomenon has also been validated in patients with RCC and tumor thrombus [23, 24]. The paradox of the prognostic value of BMI may be partly explained by the need for extra energy to battle cancer and the endocrine function of adipose tissue [25].

Here, our study first reveals the relationship between BMI and the need of IVC resection during thrombectomy for RCC tumor thrombus. This is true in Mayo level I–IV patients and is also true when we narrowed the cohort to Mayo levels II–IV, a group that has a higher possibility of IVC resection and a greater need for risk stratification. We defined any resection of IVC wall as the outcome of interest, which is different from previous studies [9–12]. A preoperative prediction model proposed by the Mayo Clinic defined the primary endpoint as the resection of the IVC that resulted in the need of vascular reconstruction beyond primary cavorraphy [11]. The subsequent external validation study of this model adopted the same endpoint but failed to validate the model’s power in a 37-case cohort [12]. It is not difficult to understand this contradiction if we take into account the possibly different reconstruction criteria between these institutions. The lack of a specific consensus on vascular reconstruction criteria might limit further generalizability of this prediction model. Other studies defined pathologic invasion of the wall of the IVC or the renal vein as the primary outcome of interests [9, 10]. Pathologic invasion as the primary endpoint is more objective but also has its own drawbacks. It might overlook other factors resulting in the resection of the IVC wall, such as tight adherence or bland thrombus. From this point of view, we chose the need of any resection of IVC wall as the endpoint to represent the difficulty of thrombectomy. Resection might result from the direct invasion of the tumor thrombus, tight adherence between the thrombus and the venous wall, or a bland thrombus that cannot be dissected. No matter which reason, it increases the difficulty of the surgery and harbors the need of complicated reconstruction. Defining the risk factors of this endpoint can help surgeons to stratify patients preoperatively, determine which patient warrants reconstruction planning, and lower the risk of one of the most complicated urologic surgeries.

We noticed a correlation between BMI and the length (PCC = 0.23, *P* = 0.037) and width (PCC = 0.27, *P* = 0.014) of the tumor thrombus. This could partly explain why BMI is positively related to the IVC resection rate. Furthermore, the tendency of comorbidity of hypertension and diabetes (*P* = 0.006 and 0.012 respectively), which are common vascular risk factors, may also contribute to tumor thrombus adherence or distal bland thrombus formation, which in turn increases the IVC resection rate in high-BMI patients.

The resection rate (51.2%) was high in our cohort. As mentioned before, we calculated the rate by any resection of the IVC wall; thus, it is natural that the rate is higher compared to the rate in the study, which only counted resections requiring complicated reconstruction (22%) [11], or another study, which only counted the IVC interruption (25%) [26]. We also noticed that BMI (median 23.1 kg/m^2^, interquartile ranges [IQRs] 21.1–26.1 kg/m^2^) is apparently lower in our cohort compared to western cohorts (median 26.5–29.0 kg/m^2^) [23, 27, 28]. It is easy to understand this difference because of the general low BMI of Asian populations [29], but it warrants further external validation of our finding in western cohorts.

In univariate analysis, we also found primary tumor diameter, tumor thrombus width, VTT level, and IVC occlusion were associated with the resection of IVC. Though not significant after adjustment for BMI, these factors should also be considered in risk stratification. It is worth mentioning that primary tumor diameter is negatively related to the need for IVC resection (univariate analysis, *P* = 0.022); although this needs further validation, it at least proves that a large primary tumor should not be considered as a regular risk factor for IVC resection.

There are also several limitations in our study. The need for resection of the IVC lacks postoperative pathological verification due to the pathological sampling problem. However, as mentioned before, since our aim was to predict the difficulty of surgery, pathology is not a necessary endpoint. Additionally, the present study is limited by its retrospective and single-center nature. Prospective study and external validation is needed in the future.

In conclusion, we identified BMI as an independent risk factor for the need for IVC resection during thrombectomy of RCC with tumor thrombus. More careful preoperative preparation for the vascular resection and/or reconstruction is warranted in patients with a higher BMI. Further external validation is needed in western cohorts, which have higher overall BMI than our cohort.

## Abbreviations

AUC: Area under the curve
BMI: Body mass index
CI: Confidence interval
c-index: Concordance index
IQRs: Interquartile ranges
IVC: Inferior vena cava
OR: Odds ratio
PCC: Pearson’s correlation coefficient
RCC: Renal cell carcinoma
ROC: Receiver operating characteristic
SCr: Serum creatinine
VTT: Venous tumor thrombus
